# Heterozygous LRP1 deficiency causes developmental dysplasia of the hip by impairing triradiate chondrocytes differentiation due to inhibition of autophagy

**DOI:** 10.1073/pnas.2203557119

**Published:** 2022-09-06

**Authors:** Wenjin Yan, Liming Zheng, Xingquan Xu, Zheng Hao, Yibo Zhang, Jun Lu, Ziying Sun, Jin Dai, Dongquan Shi, Baosheng Guo, Qing Jiang

**Affiliations:** ^a^State Key Laboratory of Pharmaceutical Biotechnology, Division of Sports Medicine and Adult Reconstructive Surgery, Department of Orthopedic Surgery, Nanjing Drum Tower Hospital, The Affiliated Hospital of Nanjing University Medical School, Nanjing 210008, Jiangsu, PR China;; ^b^Branch of National Clinical Research Center for Orthopedics, Sports Medicine and Rehabilitation, 210008, PR China;; ^c^Nanjing Zhongyangmen Community Health Service Center, Kang'ai Hospital, Nanjing, 210037, China

**Keywords:** developmental dysplasia of the hip, low-density lipoprotein receptor-related protein, autophagy, cartilage development

## Abstract

Developmental dysplasia of the hip (DDH) is one of the most common congenital skeletal malformations; however, its etiology remains unclear. Here, we conducted whole-exome sequencing in eight DDH families followed by targeted sequencing of 68 sporadic DDH patients. We identified likely pathogenic variants in the *LRP1* (low-density lipoprotein receptor-related protein 1) gene in two families and seven unrelated patients. All patients harboring the *LRP1* variants presented a typical DDH phenotype. The heterozygous *Lrp1* knockout (KO) mouse (*Lrp1*^+/−^) showed phenotypes recapitulating the human DDH phenotypes, indicating *Lrp1* loss of function causes DDH. *Lrp1* knockin mice with a missense variant corresponding to a human variant identified in DDH (*Lrp1^R1783W^*) also presented DDH phenotypes, which were milder in heterozygotes and severer in homozygotes than those of the *Lrp1* KO mouse. The timing of triradiate cartilage development was brought forward 1 or 2 wk earlier in the LRP-deficient mice, which leads to malformation of the acetabulum and femoral head. Furthermore, *Lrp1* deficiency caused a significant decrease of chondrogenic ability in vitro. During the chondrogenic induction of mice bone marrow stem cells and ATDC5 (an inducible chondrogenic cell line), Lrp1 deficiency caused decreased autophagy levels with significant β-catenin up-regulation and suppression of chondrocyte marker genes. The expression of chondrocyte markers was rescued by PNU-74654 (a β-catenin antagonist) in an shRNA-Lrp1–expressed ATDC5 cell. Our study reveals a critical role of LRP1 in the etiology and pathogenesis of DDH, opening an avenue for its treatment.

## Significance

Developmental dysplasia of the hip (DDH) is one of the most common congenital skeletal malformations; however, its etiology remains unclear. Here, we conducted whole-exome sequencing and identified likely pathogenic variants in the *LRP1* (low-density lipoprotein receptor-related protein 1) gene in two families and seven unrelated patients. We found that the timing of triradiate cartilage development was brought forward 1 or 2 wk earlier in the LRP-deficient mice, which leads to malformation of the acetabulum and femoral head. Furthermore, *Lrp1* deficiency caused a significant decrease of chondrogenic ability in vitro. Our study reveals a critical role of LRP1 in the etiology and pathogenesis of DDH, opening an avenue for its treatment.

Developmental dysplasia of the hip (DDH; MIM 142700) is one of the most common congenital malformations of the skeleton that affects 0.56 to 3.8‰ of newborns. DDH covers a spectrum of hip disorders ranging from mild dysplasia to irreducible dislocation (1, 2). DDH is a common cause of osteoarthritis (OA) of the hip joint. Hence, understanding of its etiology and pathogenesis will lead to treatment of OA. Numerous DDH candidate genes and loci have been identified by case-control and genomewide association studies; however, few associations have been replicated in independent populations (3, 4). Common polymorphisms account for only a small amount of DDH heritability with relatively a low rate of replication, especially among different ethnic groups (5). Some DDH patients are reported to exhibit autosomal dominant inheritance, but their causal genes are unknown (6, 7). Thus, the genetic basis of DDH needs more studies.

Anatomically, DDH is defined as the mismatch of the femoral head and acetabulum. The acetabula of DDH patients show a significantly decreased volumes compared to the normal acetabula (8). The decrease of acetabular volumes increases local stress on the articular surface and the instability of the hip joint, causing pain and disability of the hip joint and eventually OA. Development of an acetabulum depends on the hemispherical acetabular cartilage and the Y-shaped triradiate cartilage. The acetabular cartilage deepens the acetabulum due to interstitial growth and peripheral deposition. The triradiate cartilage increases the height and width of the acetabulum by bidirectional growth, resulting in increases of acetabular surface area and volume (9). However, the molecular mechanism controlling acetabular development is poorly understood.

Emerging evidence reveals that the low-density lipoprotein receptor-related protein (LRP) family plays key roles in skeletal development. Various LRP5 loss-of-function (LoF) mutations (point, frameshift, and nonsense mutations that disrupt WNT/β-catenin signaling) have been linked to osteoporosis pseudoglioma (10). Lrp6, Lrp4, Lrp2, Lrp8, and Lrp1 are known to play a role in bone and cartilage homeostasis (11–14). Mutations in LRP6 cause tooth agenesis (15, 16). Mutations in human LRP4 have been associated with bone diseases such as osteoporosis and sclerosteosis (17, 18). Increased bone formation and decreased bone resorption were observed in both mr-Lrp4^mitt^ and Lrp4^Ocn^-cko mice (19). Mutations in LRP2 have been shown to cause Donnai-Barrow syndrome or facio-oculo-acoustico-renal syndrome, a syndrome associated with facial dysmorphism (20, 21). LRP8 has also been shown to be an activator of Wnt/β-catenin signaling during osteoblast differentiation (22). However, no association between genetic variation within the human LRP8 and LRP1 locus and skeletal abnormalities has been reported so far.

LRP1 is a transmembrane endocytosis receptor involved in a variety of cellular processes, such as proliferation, differentiation, and metabolism. *LRP1* variants have been reported to be associated with decreases in bone mineral density and bone mineral content (23). LRP1 is expressed at a high level in articular cartilage chondrocytes and protects the matrix in joints by suppressing cartilage-degrading enzymes (24, 25). LRP1 enhances the Wnt signaling pathway, which participates in multiple developmental process, including embryonic development, tissue homeostasis, cell proliferation, and differentiation (26). Canonical Wnt signaling converges on the accumulation and translocation of β-catenin into the nucleus (27). LRP1 is associated with autophagy, which plays an important role in cell survival and differentiation (28, 29). LRP1 acts as a positive mediator in the activation of autophagy (30–32).

In this study, we identified eight DDH families of likely autosomal dominant inheritance. Using whole-exome sequencing (WES), we analyzed the families and identified *LRP1* as a candidate gene for DDH. We then sequenced 68 unrelated DDH subjects using targeted sequencing and identified seven subjects with likely pathogenic *LRP1* variants. A knockin (KI) mouse of an *Lrp1* missense variant corresponding to a human variant identified in DDH and an *Lrp1* knockout (KO) mouse mimicked human DDH phenotypes. By using the DDH model mice with *Lrp1* deficiency, we discovered a critical role of LRP1 in the development of the Y-shaped triradiate cartilage. We also discovered that LRP1 deficiency decreased chondrogenic ability through inhibition of autophagy level associated with β-catenin up-regulation. The decreased chondrogenesis caused by LRP1 deficiency was rescued by the treatment of PNU-74654 (PNU; a β-catenin antagonist) in ATDC5 cells transfected with short hairpin RNA (shRNA)-Lrp1, which illustrated that β-catenin inhibition could be a therapeutic target for decreased chondrogenic ability caused by LRP1 deficiency.

## Methods

### Study participants.

**Fig. 1.**
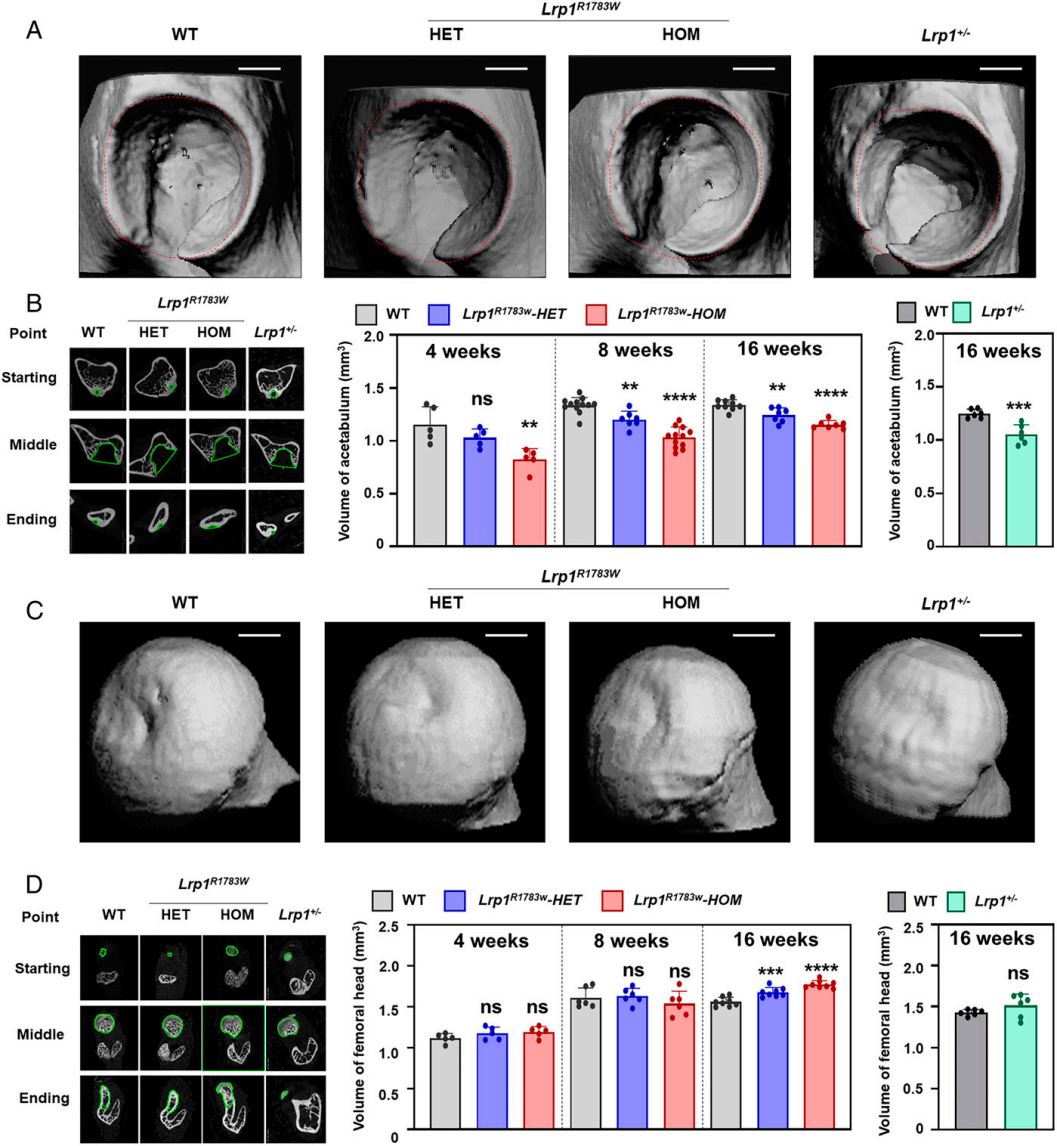
3D micro-CT images of the DDH phenotype of heterozygous and homozygous *Lrp1^R1783W^* mice, *Lrp1*^+/−^ mice, and their WT littermates at 8 wk. (*A*) Micro-CT images of the acetabulum. (*B*) The volume of the acetabulum measured according to the ROI. (*C*) Micro-CT images of the femoral head. (*D*) The volume of the femoral head. Volumes were measured according to the ROI. Scale bar: 1 mm. Values = means ± SD; ns, not significant; **P < 0.01; ***P < 0.001; ****P < 0.0001; HET, heterozygote; HOM, homozygote.

A cohort of 17 DDH patients from eight families and another cohort of 68 unrelated DDH patients were enrolled in Kang’ai Hospital in China. All individuals in this study were Han-Chinese and presented with a typical DDH phenotype accompanied by complete or incomplete dislocation of the hip joints. Clinical examinations and anterioposterior radiographs of the pelvis in the supine position (*SI Appendix*, Fig. 1) were evaluated by three orthopedic surgeons. This study was approved by the Hospital Medical Ethics Committee of Nanjing Drum Tower Hospital (Jiangsu, China). Signed informed consent was obtained from all subjects participating in the study.

### WES.

Genomic DNA was extracted from the peripheral blood of the patient and family members using a Qiagen DNA Mini kit (Qiagen, Hilden, Germany). The DNA was divided into smaller fragments of 200 to 250 bp using an ultrasonic instrument (Covaris LE220, Covaris, Woburn, MA) and purified with Ampure beads (Beckman Coulter, Brea, CA) to add a poly(A)/joint reaction to the end of the purified DNA fragments. The gene-trapping chip (Roche NimbleGen, Madison, WI) was hybridized with the purified DNA fragments. Following hybridization, captured DNA fragments were sequenced using an Illumina HiSeq2500 analyzer (Illumina, San Diego, CA) and read using Illumina Pipeline software (version 1.3.4). Burrows–WheelerAligner (BWA version 0.59) (33) was used to align sequence reads to the human genome reference (build 37). Duplicated reads were removed from subsequent analyses. Sequence variants were identified via comparisons with the national center for biotechnology information (NCBI) reference sequence NM 005529.5 and annotated by the current version of ANNOVAR (July 16, 2017; https://annovar.openbioinformatics.org/en/latest/) with information from Online Mendelian Inheritance in Man (OMIM), Gene Ontology, the Kyoto Encyclopedia of Genes and Genomes (KEGG) Pathway, MutationTaster, the 1000 Genomes Project, ExAC, and gnomAD (34–38). The variants with read depths of less than 4× were filtered out according to the Genome Analysis Toolkit (39).

### Bioinformatics analysis of variants.

According to previous pedigree analyses, DDH mainly follows autosomal dominant inheritance (40, 41). Therefore, we focused on dominant variants in WES. Considering the reported incidence of DDH in China (42, 43), genetic variants with allele frequencies ≥0.005 in the human population genome datasets (ExAc_EAS, 1000g_chbs, ESP6500, and gnomAD) were filtered out. Missense variants predicted to be damaging simultaneously by the bioinformatics tools of Sorting Intolerant From Tolerant (SIFT) and/or MutationTaster, ClinPred, and Domain Adaptive Neural Network (DANN) scores were preferred for further evaluation (44–47), while no nonsense, frameshift, or essential splice-site variants were found.

### Generation of mouse models.

**Fig. 2.**
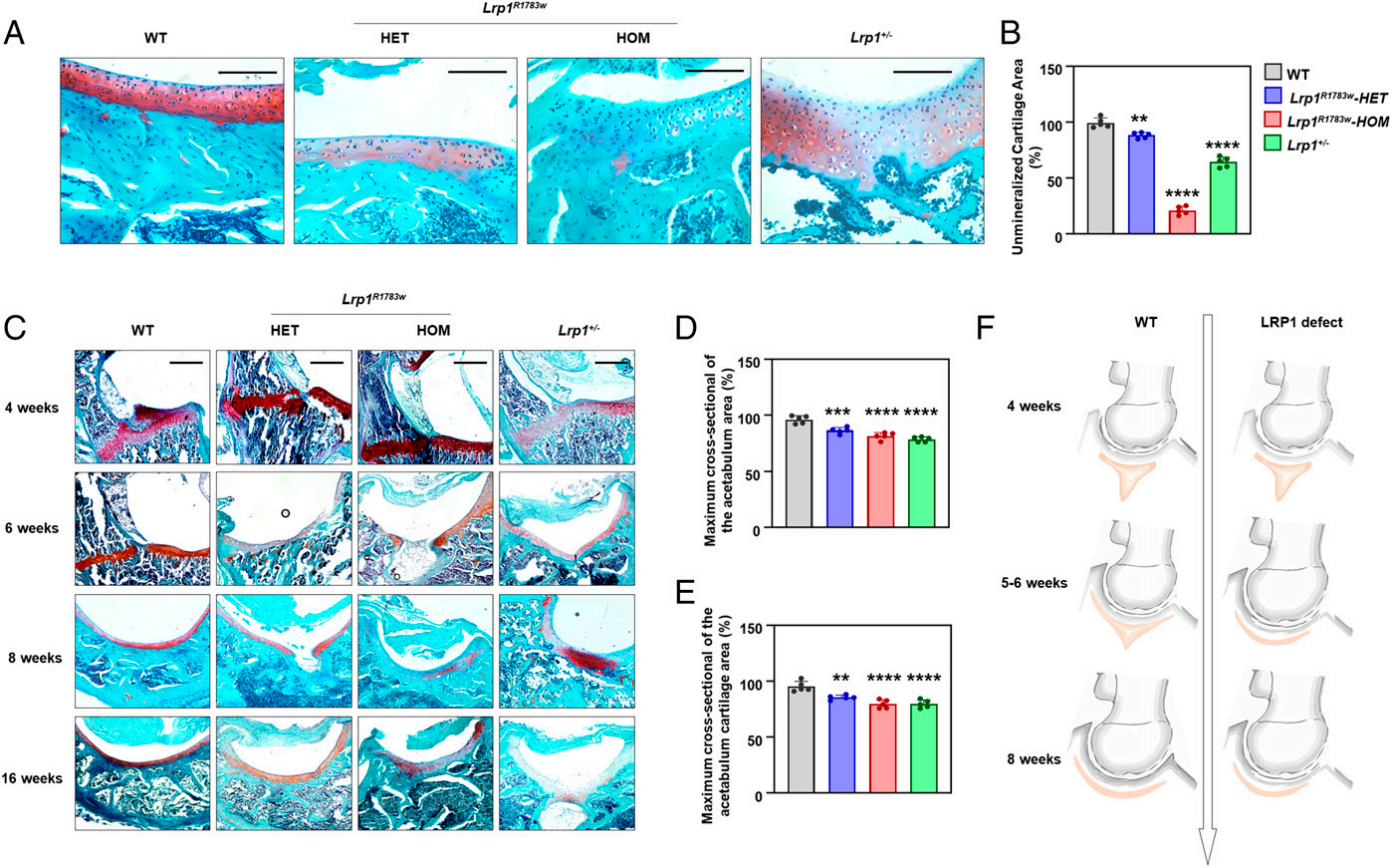
Histology of the hip joints of heterozygous and homozygous *Lrp1 ^R1783W^* mice, *Lrp1*^+/−^ mice, and their WT littermates. (*A*) Sections of mice acetabulum cartilage at 40x magnification at 8 wk. Scale bar: 100 μm. *Lrp1^R1783W^* and *Lrp1*^+/−^ mice showed sparser acetabulum cartilage compared with WT mice. (*B*) The unmineralized cartilage area calculated using the contrast red-green feature. Values = means ± SD; *P < 0.01; **P < 0.0001. The area of WT mice was significantly larger than those of *Lrp1 ^R1783W^* and *Lrp1*^+/−^ mice. (*C*) Sections in 10x magnification at 4, 6, 8, and 16 wk. Scale bar: 200 μm. The triradiate cartilage had closed completely before 6 wk in KI and KO mice, while it closed after 8 wk in WT mice. The quantitative results of maximum cross-sectional of the actabulum area (*D*) and maximum cross-sectional of the acetabulum cartilage area (*E*) was shown. (*F*) Schematic of the LRP1-deficient hip joint. Premature fusion of the triradiate cartilage (red) causes dysplasia of the hip.

To test the role of *LRP1* for DDH in vivo, we established by CRISPR-Cas9 editing a mouse KI line, *Lrp1^R1783W^* (48, 49). The mouse variant, c.5350C > T: p.1784R > W. NM_008512.2) corresponds to a human *LRP1* variant, c.5347C > T: p.R1783W (NM_002332), which we identified in a DDH family (*SI Appendix*, Fig. 2*A*). The single-guide RNA (sgRNA) was specifically designed against Lrp1-Mus musculus: NM_008512.2 to the position of *Lrp1* c.5350C. The corresponding sgRNA ligated to pX458 plasmid was transfected into embryonic stem cells (ESCs). Single colonies were expanded for genotyping after 24 h. The ESCs carrying genetic modifications were injected into blastocysts, which were generated by mating superovulated female mice with wild-type (WT) C57BL/6 male mice. After a short in vitro culture, the injected blastocysts were transferred into pseudopregnant female mice. The *Lrp1* variant was identified in founder mice and their offspring using PCR and Sanger sequencing. We also achieved *Lrp1* KO mice (*Lrp1*^+/−^) during the establishment of the *Lrp1* KI mice. The KO mice presented the frameshift deletion of *Lrp1* around c.5350 (NM_008512.2: c.5350_5354del: p.R1784fs*51).

All animal experiments were approved by the Institutional Animal Care and Use Committee (permit 2019AE01118) of Nanjing Drum Tower Hospital (Jiangsu, China). All animal experiments were carried in accordance with the relevant guidelines and regulations.

### Microcomputed tomography (micro-CT).

Micro-CT (Scanco Medical, Bassersdorf, Switzerland) reconstructions of the acetabulum and femoral head of each mouse were conducted, and the volumes of the acetabulum and femoral head were measured accordingly. The scanner indexes were set at a current of 177 μA, a voltage of 45 kVp, and a voxel size of 15.6 μm. Three-dimensional (3D) analysis of acetabular orientation used a semiautomated algorithm (50). The volume of acetabular fossa was determined according to region of interest (ROI) using ImageJ freeware by measuring the edge of the acetabulum and femoral head (51).

### Cell culture.

Bone marrow stem cells (BMSCs) were obtained from tibias and femurs of *Lrp1*^+/−^ and WT mice (4 wk old). The mice were sacrificed by cervical dislocation after anesthesia and sterilized using 75% ethanol for 10 min before dissection. After dissecting the metaphyseal ends under sterile conditions, the bone marrow cells were flushed out using Dulbecco’s modified Eagle’s medium (DMEM)/F12 medium and centrifuged at 1,000 rpm for 5 min. BMSCs were culture expanded using the MesenCult Expansion Kit (Mouse) (catalog 05513, Stemcell Technologies). The chondrogenic differentiation was induced by using the differentiation media (catalog HUXMA-90041, Cyagen Bioscience, Guangzhou, China). In addition, a pellet consisting of 5 × 10^5^ BMSCs was cultured in a 15-mL microfuge tube for 4 wk with the above chondrogenesis differentiation media.

ATDC5 cells, a chondrocyte progenitor cell line, were cultured in DMEM/F12 medium supplemented with 5% FBS, 100 U/mL penicillin, and 100 μg/mL streptomycin. Cells were subcultured at 70 to 80% confluence using differentiation medium, which was identical to the maintenance medium with the addition of 1% Insulin-transferrin-sodium selenite (ITS). The differentiation medium was changed every other day from day 2 to day 7. For micromass culture, 5 × 10^5^ ATDC5 cells were seeded at the center of the 24-well plate, avoiding touching the sides of the wells. The chondrogenic differentiation medium was supplemented with 10 ng/mL Transforming growth factor-beta 1 (TGF-β1) and maintained for 21 d.

All cells were cultured in a humidified incubator with 95% air and 5% carbon dioxide at 37 °C, and the medium was refreshed twice per week.

### Histologic analysis.

For immunostaining, the hip joints and cell pellets generated from BMSCs of the mice were fixed in 4% (vol/vol) paraformaldehyde and decalcified at 4 °C. The hip joints for histological staining were embedded in paraffin, sectioned, and sliced into 3-μm sagittal sections. BMSC pellets were dehydrated with sucrose and embedded in optimal cutting temperature compound (OCT) followed by CryoJane frozen sections. Samples were stained with Safranin O/fast green (Sigma-Aldrich) or Alcian blue (Sigma-Aldrich) as previously described (52). The unmineralized cartilage area stained by Safranin O was measured by two blinded authors (L.Z. and Y.Z.) using the contrast red-green feature as previously described (53). ImageJ (version 1.8.0) was used to measure and calculate the maximum cross-section of the acetabulum area and the cartilage area in 16-wk-old mice.

### Immunohistochemistry and immunofluorescence.

After dehydrating in an ethanol gradient and clearing with dimethylbenzene, 3% hydrogen peroxide was used to rinse sections. Sections were blocked with goat serum (Gibco) for 1 h at 37 °C and incubated with primary antibodies overnight at 4 °C after antigen retrieval by 0.1% pepsin (Sigma). Immunohistochemistry sections were incubated with horseradish peroxidase (HRP)–conjugated secondary antibody (Biosharp) for 1 h at 37 °C, and 3,3-diaminobenzidine was used to visualize the positive cells. Images were photographed under a Leica light microscope. Immunofluorescence sections were incubated with fluorescein isothiocyanate (FITC)- or TRITC-conjugated secondary antibodies for 1 h at 37 °C, and then the nucleus was stained with 4,6-diamidino-2-phenylindole (Abcam) for 4 min. The images were obtained by an Olympus FV-1000 fluorescence confocal microscope. Images used for comparisons of different treatments were analyzed by ImageJ software on the same instrument settings and exposure times and were processed consistently in the same way. The percentages of immune-positive cells for SRY-box transcription factor 9 (Sox9) and β-catenin–positive area were calculated. The relative fluorescence intensities (as percentages) for Collagen Type II Alpha 1 Chain (Col2A1), light chain 3B (LC3B), and β-catenin–positive area were calculated.

### iTRAQ proteome experiment.

An Isobaric tags for relative and absolute quantitation (iTRAQ) proteome experiment was conducted by Shanghai Bioprofile Technology Company. Briefly, peptides were labeled with Tandem Mass Tag (TMT) reagents according to the manufacturer’s instructions (Thermo Fisher Scientific, Logan, UT). Then, a TMT-labeled peptide mixture for nano-LC-MS/MS analysis was performed as previous described (54). The resulting liquid chromatography tandem mass spectrometry (LC-MS/MS) raw files were imported into MaxQuant software (version 1.6.0.16) for data interpretation and protein identification against the database UniProt_Hordeum-vulgare_201747-20180125 (downloaded on January 25, 2018, including 201,747 protein sequences), which was sourced from the protein database at https://www.uniprot.org/uniprot/?query=Hordeum+vulgare&sort=score.

### Lrp1 knockdown by shRNA in ATDC5 cells.

*Lrp1* was specifically knocked down in ATDC5 cells using concentrated lentiviruses expressing *Lrp1*-shRNA purchased from SyngenTech (Beijing, China). Lentivirus complexes were prepared (50 multiplicity of infection), and transfection was performed in ATDC5 cells. A total of 5 × 10^6^ cells were used for each transient transfection using standardized protocols. Twenty-four hours after transfection, cells were screened by puromycin for 2 d. The transected cells were lysed, and the Lrp1 expression level was assayed by western blot. Polyclonal lines were expanded and treated with puromycin for an additional 5 d before banking.

### Inhibition of β-catenin by PNU in ATDC5 cells.

The PNU compound is a non-Food and Drug Administration proved drug which prevents Tcf from binding to β-catenin, acting as a Wnt/β-catenin antagonist (55). β-catenin levels in ATDC5 cells were observed after 7 d of treatment with 10 and 20 μM PNU in a chondrogenic differentiation medium (catalog HUXMA-90041, Cyagen Bioscience, Guangzhou, China).

### Inhibition of autophagic flux by bafilomycin A1 in ATDC5 cells.

The estimation of autophagic flux is important to distinguish increased autophagosome formation from impaired degradation. To measure autophagic flux, ATDC5 cells were incubated with the indicated drugs in the presence or absence of the bafilomycin A1 (BFL-A1; Abcam, ab120497), an inhibitor of autophagosome-lysosome fusion in vitro (56). BFL-A1 was used at a concentration of 1 nM. Cells were grown in DMEM/F12 medium with 10% FBS at 37 °C, in a 5% carbon dioxide incubator. Experimental cultures were initiated by culturing at a density of 2 × 10^6^ cells/mL and sampled at the indicated times for 4 h. Western blot analysis and immunofluorescence staining using mouse anti-LC3B (Cell Signaling Technology, Boston, MA, 83506) and rabbit anti-Lamp1 (Cell Signaling Technology, 9091S) antibody were performed.

### Western blotting.

Total proteins were extracted from the mice acetabulum and cells (BMSCs and ATDC5 cells). Total proteins were lysed with Radio Immunoprecipitation Assay lysis buffer [50 mM Tris⋅HCl (pH 7.4), 150 mM NaCl, 1 mM EDTA, 0.5% sodium deoxycholate, 1% Nonidet P-40, and 0.1% sodium dodecyl sulfate (all from Cell Signaling Technology)] supplemented with inhibitors of protease (539134, Calbiochem, San Diego, CA) and phosphatase (524625, Calbiochem). Nuclear and cytoplasmic protein extractions were performed using NE-PER nuclear and cytoplasmic extraction reagents (Thermo Fisher Scientific), sufficient reagents for extracting 50-cell pellet fractions with packed cell volumes of 20 µL each (a total of ∼2 g of cell paste). Proteins were separated in 10% sodium dodecyl sulfate polyacrylamide (SDS-PAGE) and transferred onto polyvinylidene fluoride membranes, which were blocked for 2 h at room temperature using 4% nonfat milk–Tris buffered saline Tween solution and incubated overnight at 4 °C with specific antibodies. Proteins were detected using goat anti-rabbit HRP-labeled secondary antibodies (Fude Biological Technology, Hangzhou, China) for 2 h at room temperature. Membranes were scanned and the results were quantified using the Tanon-5200 system (Biotanon, Shanghai, China). Antibodies were as follows: primary antibodies against RUNX family transcription factor 2 (Runx2), Lrp1, nuclear pore glycoprotein p62 (p62), mitochondrial fission 1 protein (Fis1), protein kinase R–like endoplasmic reticulum kinase (Perk), Sox9, collagen type I (Col1), collagen type II (Col2), collagen X (Abcam), anti-LC3-I/II antibody (Sigma-Aldrich), Beclin1, AKT serine/threonine kinase (Akt), p-akt, mTOR, p-mTOR, GAPDH antibody (Proteintech), Erk1/2, pErk, GSK-3β, p GSK-3β, Lrp6, Dvl2, Lamp1 (Cell Signaling Technology), and rabbit second antibody (Biosharp, Hefei, China).

### Real-time RT-PCR.

Total RNAs from murine chondrocytes and ATDC5 cells were extracted using TRIzol reagent (Thermo Fisher Scientific). Reverse transcription (RT) was performed using the PrimeScript RT Reagent Kit (Takara, Kyoto, Japan). RT-PCR was performed using SYBR green PCR Master Mix (Vazyme Biotech, Nanjing, China) in a LightCycler 480-II (Roche, Mannheim, Germany). The relative expression of each gene was defined using the 2^2^ΔΔCt (relative quantitative method) method. All data were normalized to the expression of the β-actin gene. All reactions were performed in triplicate.

### Statistical analysis.

All data were expressed as means ± SDs. Multiple comparisons of data among the groups were assessed by one-way ANOVA followed by the least significant difference test (Fisher test). Significance was evaluated by the unpaired Student’s *t* test for comparisons between two groups. The Kruskal-Wallis test was used to detect significant differences among samples. The Mann–Whitney *U* test (post hoc test) was used to compare the volume differences between the mutant group and the control group. The SPSS software program (version 10.0, SPSS, Chicago, IL) was used for statistical analyses.

## Results

### Identification of *LRP1* variants in DDH patients.

We collected 17 DDH patients in eight families (familial group) and 68 sporadic DDH patients (nonfamilial group) who had severe DDH with dislocation of the hips. WES was performed on the familial group. Two rare *LRP1* variants, c.5347C > T [p.R1783W] and c.6386C > A [p.T2129K] (NM.002332), were identified in two families (Table 1 and *SI Appendix*, Fig. 2*A*). The patients were heterozygous for the missense variants. Amino acid alignments around the missense variants were highly conserved among different species (*SI Appendix*, Fig. 2*B*).

**Table 1.** *LRP1* variants in patients in two DDH families and 68 sporadic DDH patients

Gene region	Nucleotide	Amino acid	Allele frequency				In silico prediction				Patient No.
			1000g_chbs	ExAC_EAS	ESP6500	gnomAD	SIFT	MutationTaster	ClinPred	DANN score	
Exon 40	c.6386C > A	p.T2129K	–	0.0006	–	2.784E-05	D	D	D	0.991	2726
Exon 32	c.5347C > T	p.R1783W	–	0.0001	–	2.785E-05	D	D	D	0.999	2621
Exon 81	c. 12575C > A	p.P4192Q	–	0.0001	–	1.697E-05	T	D	T	0.882	3193
Exon 74	c. 11441A > G	p.H3814R	–	–	–	–	T	D	D	0.962	3174
Exon 6	c.670C > G	p.P224A	–	–	–	–	T	D	T	0.798	3193
Exon 54	c.8600A > G	p.N2867S	–	0	–	1.196E-05	T	N	D	0.997	3210
Exon 26	c.4361 + 6C > T	splicing	0.000399	0.0038	–	0.0002538	–	–	–	–	3151
Exon 18	c.2798–4C > A	splicing	–	0.0001	–	2.023E-05	–	–	–	–	3196
Exon 42	c.6842–4A > G	splicing	0.000599	0.0024	–	0.0002133	–	–	–	–	3161
Exon 13	c.2174C > T	p.T725M	–	0.0001	–	1.595E-05	T	D	T	0.986	3161

LRP1: NM_002332. –, not covered in the database; T, tolerated; D, damaging; N, polymorphism.

We then performed the *LRP1* targeted sequencing in the nonfamilial group and identified eight rare variants in seven cases (Table 1). Five missense variants and three splice-site variants were identified. The phenotypes of the above patients with the *LRP1* variants are in *SI Appendix*, Table 1 and Fig. 1. The variants were predicted to be likely damaging to the LRP1 protein function by missense prediction tools. The probability of being LoF intolerant of *LRP1* is 1 (https://gnomad.broadinstitute.org), meaning that *LRP1* is definitely a LoF-intolerant gene. A previous study showed that LoF-intolerant genes include virtually all known severe haploinsufficient disease genes, which points to genes in which heterozygous LoF confers some nontrivial survival or reproductive disadvantage (57).

### *Lrp1*^R1783W^ is a LoF allele.

**Fig. 3.**
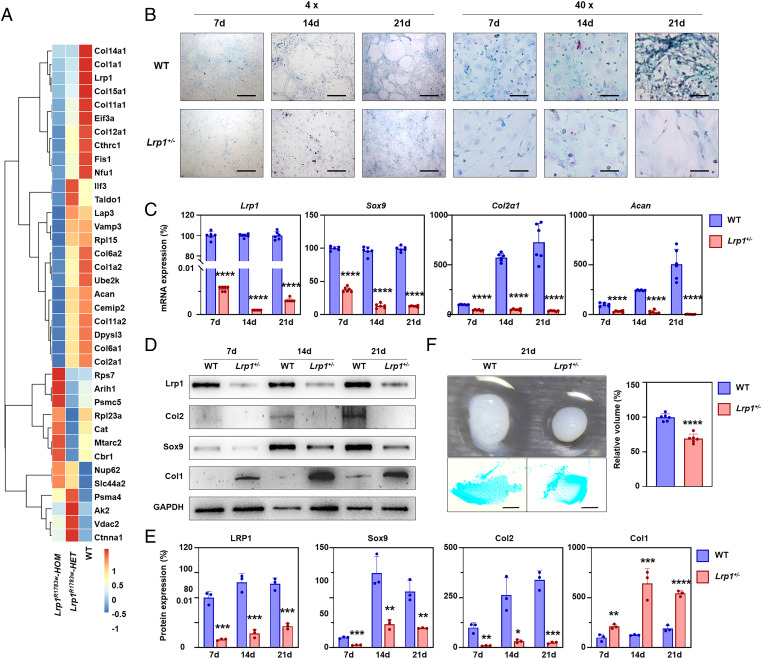
LRP1 deficiency decreases chondrogenic ability. (*A*) Expression of chondrocyte and autophagy marker proteins in the hip joint of heterozygous and homozygous *Lrp1^R1783W^* mice detected by iTRAQ. (*B*) Alcian blue staining showing chondrogenic potential of BMSCs induced by chondrogenic differentiation medium for 7, 14, and 21 d. Scale bar for 4x: 500 μm. Scale bar for 40x: 50 μm. (*C*) qPCR assays for *Lrp1, Col2, Sox9,* and *Acan* in BMSCs at 7, 14, and 21 d after chondrogenic induction. The relative expression was normalized to *GAPDH* (*n* = 3). Three independent assays were performed. *P < 0.0001. (*D*) Western blot for Lrp1, Col2, Sox9, and Col1 in induced BMSCs for 7, 14, and 21 d. GAPDH was used as a loading control. Three independent assays were performed. (*E*) Relative protein levels determined by density analysis. Expressions of Col2 and Sox9 were decreased in KO cells while that of Col1 was increased. Values = means ± SD; *P < 0.05; **P < 0.01; ***P < 0.001; ****P < 0.0001. (*F*) 3D pellets of Lrp1^+/−^ cells and Alcian blue staining of the slice pellets. Pellets from Lrp1^+/−^ cells are smaller and had a hollow inside. Three independent experiments were performed.

To investigate the function of *LRP1* and its variants identified in DDH in vivo, we engineered a mouse KI line with *Lrp1* c.5347C > T [p.R1783W] via the CRISPR-Cas9 genome editing system. Heterozygous and homozygous KI mice with an *Lrp1*^R1783W^ allele (*SI Appendix*, Fig. 3*A*) were established. We also engineered *Lrp1* KO mice and obtained heterozygous KO mice (*Lrp1*^+/−^). Homozygous KO mice were not obtained. Blastocysts homozygous for the null allele are reported to fail to implant into the mouse uterus or to complete the implantation process (58). Western blot and protein mass spectrometry analyses showed significant decreases in expression levels of LRP1 proteins in *Lrp1*^R1783W/+^, *Lrp1*^R1783W/R1783W^, and *Lrp1*^+/−^ mice (*SI Appendix*, Fig. 3*B*). The expression level in the KI heterozygote was between those in WT and KI homozygous mice, and KI homozygous mice displayed a similar level of expression to KO mice. These results suggest that LRP1, c.5347C > T can cause LRP1 LoF with a gene dosage effect.

### *Lrp1*-defective mice display the DDH phenotype.

**Fig. 4.**
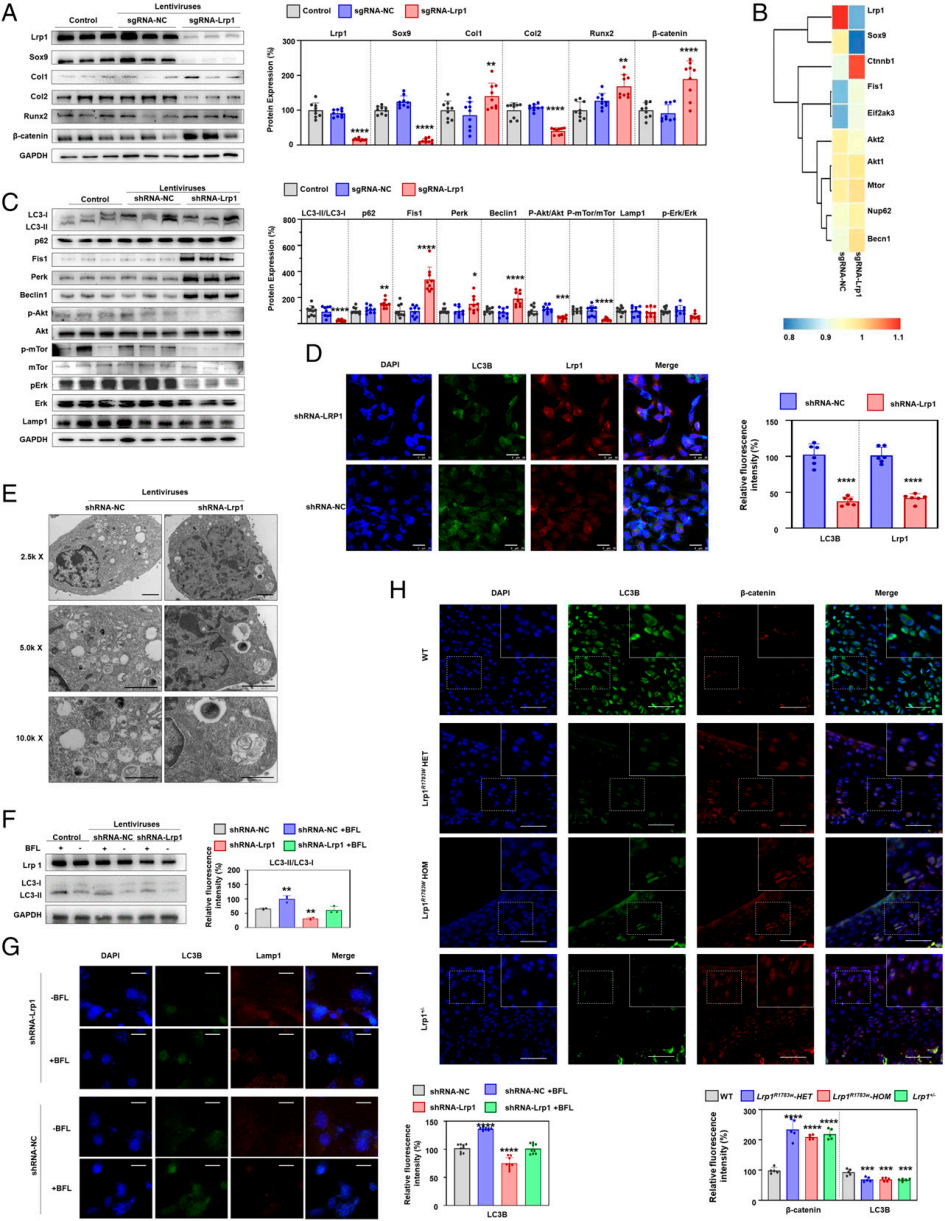
*Lrp1* knockdown reduces chondrogenic differentiation in ATDC5 cells. (*A*) Western blot for LRP1 and chondrogenic differentiation markers. The relative protein level was determined by density analysis. Results of three independent experiments (*n* = 9). Values = means ± SD; *P < 0.05; **P < 0.01; ****P < 0.0001. (*B*) Heatmaps of a protein mass spectrometry analysis of ATDC5 cells after chondrogenic induction for 7 d. (*C*) Western blot for autophagy-related proteins. (*D*) Electron micrographs showing the level of autophagy in ATDC5 cells transfected with shRNA-Lrp1 and shRNA-NC lentiviruses. Fewer autophagosomes were observed in the shRNA-Lrp1 group. Scale bar: 2 μm. (*E*) Immunohistochemistry staining of Lrp1 and LC3B in ATDC5 cells with shRNA-Lrp1 lentivirus transfection. The histological scoring system showed a decrease of LC3B in Lrp1 knockdown. (*F*) After treatment with BFL-A1, the shRNA-Lrp1 lentivirus transfection group untreated with BFL-A1 showed the lowest level of LC3II. The difference value of the LC3II level between the shRNA-NC with BFL group and the shRNA-NC without BFL group was not significant compared with the difference value of the LC3II level between the shRNA-NC with BFL group and the shRNA-Lrp1 with BFL group. (*G*) Immunofluorescence staining for autophagosome and lysosome location. LC3B-II puncta, green; LAMP2 puncta, red; DAPI, blue. Scale bar: 15 μm. (*H*) Immunohistochemistry staining of the hip sections showed increased β-catenin and decreased LC3B in heterozygous and homozygous *Lrp1 ^R1783W^* mice, *Lrp1*^+/−^ mice, and their WT littermates.

To assess the LRP1 function in hip joint development, we examined hip phenotypes of *Lrp1* KI and KO mice at 4, 8, and 16 wk. Micro-CT analyses showed a dramatic reduction of the acetabular volumes in homozygote and heterozygote KI mice and heterozygous KO mice (Fig. 1 *A* and *B*). The mouse acetabula revealed defective coverage of the femoral heads. The acetabulum volumes of the mutant mice were consistent with the Lrp1 expression levels (*SI Appendix*, Fig. 3*B*): the volume of heterozygous KI mice was greater than that of homozygous KI mice, smaller than that of WT mice, and similar to that of KI homozygous mice. In contrast, there were no differences in the volume and the shape of the femoral head among mice (Fig. 1 *C* and *D*). No differences in body weight and length were observed in 8-wk-old mice or in the general views of acetabula (*SI Appendix*, Fig. 4).

### Triradiate cartilage closes earlier in *Lrp1*-defective mice.

Sections of hip joints from 4-, 6-, 8-, and 16-wk-old mice were subjected to histological analyses. Homozygous KI and heterozygous KI mice showed similar general views of acetabula with their WT littermates (*SI Appendix*, Fig. 4) but showed sparser acetabulum cartilage compared with that of WT mice (Fig. 2*A*). The unmineralized cartilage areas of KI and KO mice were significantly decreased compared to that of WT mice (Fig. 2*B*). The triradiate cartilage had closed completely before 6 wk in KI and KO mice, while it closed after 8 wk in WT mice (Fig. 2*C*). This finding is in line with the results of micro-CT measurement of acetabulum volume. The significant difference of the timing of triradiate cartilage closure between heterozygous KI mice and their WT littermates started to appear after 8 wk. In addition, the maximum cross-section of the acetabulum area and the cartilage thickness were measured and calculated at 16 wk (Fig. 2 *D* and *E*). The acetabular section was selected to measure the area at its maximum cross-section. Statistical quantitative analysis showed that the acetabulum area and the cartilage area were significant smaller in *Lrp1*^+/−^ mice and KI mice compare with their WT littermates (*P* < 0.0001).

The hip cartilage cells of WT mice at 4 to 16 wk were round or ovoid, were in neat rows, had obvious lacuna, were well integrated with both edges, and had good column alignment. In contrast, hip cartilage cells of KI and KO mice were almost the same as fibrocartilage cells, showing irregular arrangement and no lacuna (Fig. 2*A*). According to the above observation, we assume that LRP1 deficiency might cause premature fusion of the Y-shaped triradiate cartilage and thus inhibit the bidirectional growth of triradiate cartilage and result in the miniacetabulum (Fig. 2*F*).

### Decreased chondrogenic ability of *Lrp1*^+/−^ BMSCs.

According to the iTRAQ proteome experiment results, collagen expressions in the hip joint of both heterozygous and homozygous *Lrp1^R1783W^* mice were significantly lower than those of WT littermates (Fig. 3*A*). Interestingly, autophagy-related proteins Fis1 and p62 were significantly increased in both heterozygous and homozygous *Lrp1^R1783W^* mice and presented a dosage effect (Fig. 3*A*).

To further characterize the LRP1 function at the cell level, BMSCs were isolated from WT and heterozygous KO mice and their chondrogenic potential was tested. After chondrogenic induction for 7, 14, and 21 d, their ability to differentiate into chondrocytes was verified by Alcian blue staining. Intensities of matrix proteoglycan synthesis nodules were gradually increased in a time-dependent manner. The heterozygous KO mice had fewer nodules than WT mice (Fig. 3*B*). The cell morphology of both WT and KO BMSCs exhibited normal organization and structure. We then evaluated the expression of chondrogenic differentiation markers in the BMSCs by using qPCR (Fig. 3*C*) and western blot (Fig. 3 *D* and *E*). Sox9, the early-stage differentiation marker of chondrocytes, markedly increased its expression between days 7 and 14 and maintained high levels until day 21 in both WT and heterozygous KO mice. Notably, the Sox9 expression level in *Lrp1*^+/−^ BMSCs was far lower than that in WT cells. Col2 and Aggrecan showed significant increases between days 14 and 21 in WT BMSCs, while they remained low in *Lrp1*^+/−^ BMSCs (Fig. 3 *C*–*E*). *Lrp1*^+/−^ BMSCs showed an increase of Col1 expression after 7-d differentiation (Fig. 3*E*). Thus, early chondrogenic differentiation was hampered in heterozygous KO chondrocytes.

We also induced an in vitro pellet to examine the impact of LRP1 deficiency on 3D cartilage constructs generated from BMSCs of both WT and *Lrp1*^+/−^ mice. After 21 d of induction, both WT and *Lrp1*^+/−^ BMSCs formed 3D pellets. *Lrp1*^+/−^ BMSCs showed a significant decrease in pellet size (Fig. 3*F*). Histological staining of 3D pellet cross-sections with Alcian blue showed a hollow inside the *Lrp1*^+/−^ pellet, while WT pellets showed uniform staining (Fig. 3*F*).

### LRP1 deficiency suppresses autophagic degradation of β-catenin.

**Fig. 5.**
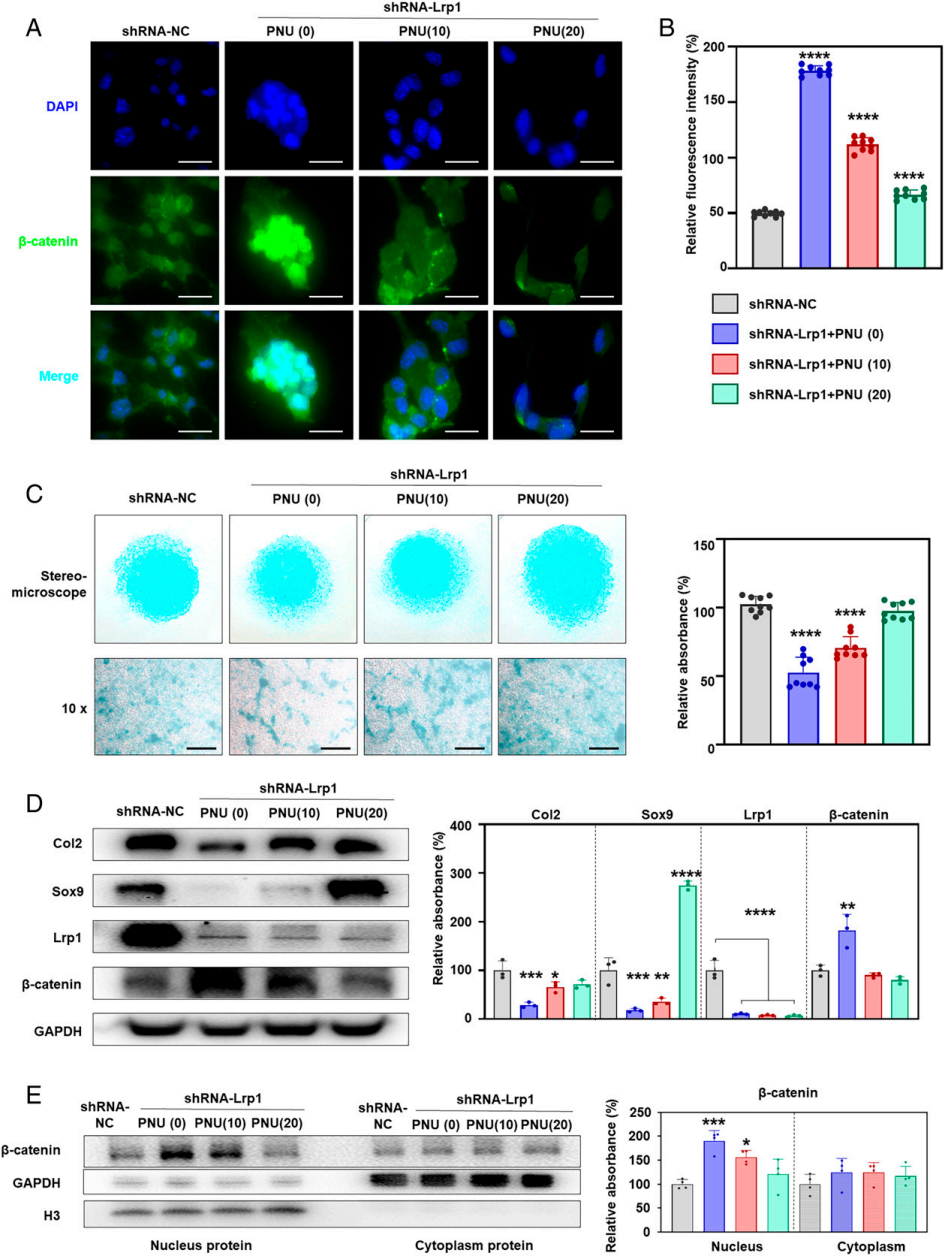
PNU rescues chondrogenic ability in LRP1-deficient ATDC5 cells. (*A*) Effects of PNU on β-catenin expression. shRNA-NC: treated with NC lentivirus. PNU (0), (10), (20): treated with sh*Lrp1* lentivirus + 0, 10, and 20 mM PNU, respectively. β-catenin, green; DAPI, blue. (*B*) Relative fluorescence intensity (as a percentage) calculated for *A*. PNU suppressed β-catenin up-regulation due to LRP1 deficiency. (*C*) ATDC5 cell micromasses after chondrogenic induction for 7 d. The lentivirus-transfected shRNA-Lrp1 cells were treated with 0, 10, or 20 μM PNU for 8 d. Chondrogenic ability was evaluated by Alcian blue staining. Effects of LRP1 deficiency on chondrogenesis were rescued by PNU. (*D*) Western blot for β-catenin, Col2, and Sox9. The lentivirus-transfected shRNA-*Lrp1* cells were treated with 0, 10, or 20 μM PNU for 8 d. Three independent experiments were assessed (*n* = 3). The relative protein level was determined by densitometry. Values = means ± SD; *P < 0.05; **P < 0.01; ***P < 0.001; ****P < 0.0001. (*E*) PNU led to a decreased level of β-catenin in both the nucleus and the cytoplasm but was much more significant in the nucleus, as detected by western blot.

To explore the molecular pathway underlying LRP1 function in cartilage development, we generated polyclonal ATDC5 cell lines by lentiviruses that expressed shRNA-Lrp1 (transfected with shRNA-Lrp1) or shRNA-NC (negative control). After the chondrogenic differentiation induced with 1% ITS for 7 d, total protein was extracted and detected by an iTRAQ proteome experiment and western blot. According to the statistical results of the iTRAQ proteome experiment and western blot verification, shRNA-Lrp1 cells showed 70% knockdown compared to the control (no treatment) and shRNA-NC cells (Fig. 4 *A* and *B*). The positive results of Alcian blue staining and negative results of Alizarin red staining indicated that the cells differentiated into chondrocytes under the induction of chondrogenic differentiation medium (*SI Appendix*, Fig. 5). Consistent with the results of animal experiments, the expression of chondrogenic differentiation markers in the shRNA-Lrp1 cells showed a significant decrease (Fig. 4*A*). Besides, the proteome experiment and western blot verification showed β-catenin was significant up-regulated in sgRNA-Lrp1 cells (Fig. 4 *A* and *B*). Interestingly, the upstream autophagy proteins Beclin1, Fis1, and Perk were significantly up-regulated in shRNA-Lrp1 cells, while mTor, Akt, Erk, and their phosphorylation levels showed significant supersession, which suggests activate autophagy **(**Fig. 4 *B* and *C*). LC3II/I, a marker for the late formation of autophagosomes, was significantly decreased, while p62 was significantly increased (Fig. 4*C*). Obviously, the accumulation of p62 showed that autophagosomes could not form. Electron microscopy showed the unique bilayer structure of autophagosomes in ATDC5, while fewer autophagosomes were observed in the shRNA-Lrp1 cells (Fig. 4*D*). Furthermore, shRNA-Lrp1 cells exhibited a reduced autophagy level, as evidenced by staining of LC3B (Fig. 4*E*). Colocalization of LC3B and Lrp1 was also showed by confocal microscopy (Fig. 4*E*). In addition, the shRNA-Lrp1 lentivirus transfection group untreated with BFL-A1 showed the lowest level of LC3II (Fig. 4 *F* and *G*). The difference value of the LC3II level between the shRNA-NC with BFL group and the shRNA-NC without BFL group was not statistically significant (*P* = 0.7178) compared with the difference value of the LC3II level between the shRNA-NC with BFL group and the shRNA-Lrp1 with BFL group, indicating that LRP1 may not affect lysosomal function (Fig. 4 *F* and *G*). Results from immunofluorescence staining revealed that BFL treatment increased the protein level of LC3B and suppressed the colocalization of LC3B-II and Lamp1 (Fig. 4*G*). These results suggest that Lrp1 is required for autophagosome formation.

Considering β-catenin’s important role in chondrogenic differentiation and its significant increase in shRNA-Lrp1 cells, we tested the relationship among Lrp1, β-catenin, and autophagy (LC3). Immunofluorescence staining of LC3B and β-catenin for slices from the hip joints of 4-wk-old mice (both heterozygous and homozygous KI, *Lrp1*^+/−^ mice, and their WT littermates) showed colocalization of LC3B and β-catenin with consistently increased β-catenin and decreased LC3B levels (Fig. 4*H*). Taken together, these data suggest that LRP1 deficiency decreases the autophagic degradation of β-catenin.

### Inhibition of β-catenin rescues the decreased chondrogenic ability caused by LRP1 deficiency in ATDC5.

As it is well known that PNU inhibits interaction between Tcf4 and β-catenin in nuclei, we examined the effect of PNU on chondrocyte differentiation in shRNA-Lrp1 ATDC5 cells. The β-catenin level was significantly up-regulated in shRNA-Lrp1 cells and was down-regulated by PNU treatment in a dose-dependent manner (Fig. 5 *A* and *B*). On the 20 μM PNU treatment, the level of β-catenin was close to that of the shRNA-NC cells (Fig. 5*B*). We evaluated the levels of total proteoglycans in the extracellular matrix (ECM) of the ATDC5 cell micromasses after chondrogenic induction for 7 d. A significant decrease in total proteoglycans was observed in shRNA-Lrp1 cells compared to shRNA-NC cells (Fig. 5*C*). The PNU treatment caused significant increases of proteoglycans (Fig. 5*C*), Col2, and Sox9 (Fig. 5*D*). PNU led to a decreased level of β-catenin in both the nucleus and the cytoplasm but was more significant in the nucleus, as detected by western blot (Fig. 5*E*), which is consist with a previous study that showed PNU may directly decrease β-catenin expression by directly binding to β-catenin in the adrenal cell line (NCI-H295) (59).

### The level of autophagy increased gradually during chondrogenesis.

To investigate the role of autophagy in chondrogenesis, ATDC5 cells were induced chondrogenic differentiation, and the levels of autophagy and β-catenin were examined by western blot on days 1, 4, 7, 10, and 12. The level of LC3II/I was significantly decreased in shRNA-Lrp1 cells compared to shRNA-NC cells, while the accumulation of p62 was significantly increased, especially on day 10, and then remained at the highest level (*SI Appendix*, Fig. 6). The levels of autophagy upstream proteins Beclin1, Fis1, and Perk were also significantly up-regulated in shRNA-Lrp1 cells (*SI Appendix*, Fig. 6). In addition, the level of β-catenin was significantly increased in shRNA-Lrp1 cells compared to shRNA-NC cells, significantly increased on day 10, and rose continuously on day 12.

## Discussion

Although some DDH patients are known to exhibit autosomal dominant inheritance (6, 7), causal genes of DDH have not been identified. *LRP1* is known as a causal gene for autosomal recessive keratosis pilaris atrophicans (60), and association with Alzheimer disease has been reported (61); however, *LRP1* has not been implicated in any skeletal diseases (OMIM 107770). The *LRP1* variants we identified in DDH were all heterozygous in the patients and cosegregated with a dominant mode of inheritance in the families. To examine the in vivo effects of *LRP1* and the variants for the development of DDH, we engineered the *Lrp1* KI mouse with the variant corresponding to one of the likely pathogenic variants (c.5347C > T; p.R1783W) and the KO mouse. The heterozygous KI mouse replicated the DDH phenotype, as did the heterozygous KO mouse, but its phenotype was milder than that of the KO mouse. Thus, our results indicate that heterozygous LRP1 LoF causes DDH and the pathogenic variant is a hypomorphic allele.

By using the mouse models, we showed that LRP1 deficiency causes DDH through early closure of the Y-shaped triradiate cartilage. During the development of the acetabulum, the Y-shaped triradiate cartilage gradually closes to form a semicircular acetabulum (62). Defects in stratification, migration, or differentiation of the triradiate cartilage can lead to abnormal acetabular volume and morphology (63, 64). The timing of the closure of the triradiate cartilage is considered crucial for acetabular dysplasia: i.e., the earlier the time of closure, the greater the chance of acetabular dysplasia (65). Our LRP1-deficient mice showed 1- to 2-wk earlier closure of the triradiate cartilage. Premature closure of the triradiate cartilage inhibits the growth of the acetabulum, resulting in the formation of the miniacetabulum of DDH (66).

We showed a decrease of chondrocyte activity and an increase of autophagy-related proteins in the hip joint cartilage of the model mice. Examination of chondrogenic differentiation markers in BMSCs from *Lrp1* KI and KO mice confirmed the decreases of their chondrogenic abilities, which led to the early closure of the Y-shaped triradiate cartilage and eventually manifested as the small acetabulum. LRP1 is known to play an important role in the activation of autophagy. The LRP1 complex triggers clathrin-dependent endocytosis and initiates signaling cascades that activate different downstream proteins correlating to autophagy (67, 68). LRP1 knockdown impairs lactoferrin-induced autophagy by completely abolishing LC3 conversion in NIH/3T3 mouse fibroblasts (69). LRP1 is also identified as the VacA receptor for toxin-induced autophagy in the gastric epithelial cell line AZ-521, and VacA internalization through binding to LRP1 regulates the autophagic process, including generation of LC3-II from LC3-I, which is involved in formation of autophagosomes and autolysosomes (70). Our studies showed a decreased autophagosome number, a decreased level of LC3II/I, and an increased level of p62 in Lrp1-deficient chondrocytes, as well as colocalization of LRP1 and LC3B, indicating that LRP1 is localized in the autophagosome structure and mediates the transformation of LC3I to LC3II.

Autophagy has not been implicated in pathogenesis of DDH to our knowledge. Autophagy helps normal degradation of chondrocyte ECM, which prevents abnormal accumulation of ECM, leading to aberrant chondrogenesis (71–74). Autophagy inhibitors 3MA and BFL significantly inhibit the activity of mouse primary chondrocytes (75). Autophagy negatively regulates the Wnt/β-catenin signaling pathway by promoting degradation of WNT pathway components DVL2 and β-catenin (76–78). More and more LRPs were found to be involved in endocytosis and in transducing signals. The late autophagy inhibitor Baf-A1 and the Wnt/β-catenin pathway inhibitor PKF115-584 reversed the effects of LRP6 on trophoblast autophagy, migration, and invasion (79). Furthermore, autophagy positively regulated the Wnt/β-catenin pathway and thus influenced osteoblastic differentiation; the Wnt/β-catenin signaling pathway could be abated by autophagy inducers but exacerbated by autophagy inhibitors (80).

Members of the LRP family regulate the Wnt/β-catenin signaling pathway (81), which is known to play a role in the differentiation processes that control the balance of osteoblasts, osteoclasts, and chondrocytes (82, 83). A highly significant boost came for the field of WNT signaling with the discovery of LRPs as WNT coreceptors for WNT/β-catenin signaling since 2000 (84–86). A series of structural studies described a clear mechanistic explanation for how the phosphorylated PPSPxS motifs on LRP5/6 impact the stabilization of β-catenin (87, 88). Lrp4, acting as a sclerostin receptor, has been demonstrated to be negatively linked with the serum sclerostin level and bone mass, thus inhibiting Wnt/β-catenin signaling and osteoblastic differentiation (89, 90). In this study, we also tested the levels of upstream proteins (p-GSK3β, Lrp6, Dvl2, and GSK3β) of β-catenin in Wnt signaling to explore whether β-catenin was up-regulated by the Wnt signaling pathway. Interestingly, the results revealed that unlike other LRPs (WNT coreceptors), p-GSK3β showed a significant decrease in the shRNA-Lrp1 treatment group and was not rescued by PNU (*SI Appendix*, Fig. 7). Meanwhile Lrp6, Dvl2, and GSK3β were up-regulated in the shRNA-Lrp1 treatment group (*SI Appendix*, Fig. 7). We assumed that the decreased level of p-GSK3β was the compensation effect of the up-regulated level of β-catenin in the cytoplasm, while the decreased p-GSK3β also caused the compensation effect to increase for Lrp6, Dvl2, and GSK3β. Considering the important roles that LRPs played in the Wnt signaling, we suggested that maybe there’s some potential interaction in the Wnt pathway among LRP1 and other LRP proteins, which would be quite interesting to explore in the future.

In conclusion, according to our study, the inhibition of autophagy led to significant up-regulation of β-catenin, which colocalized with LC3B, the autophagosome marker, suggesting that β-catenin itself is a target for clearance after autophagy activation in chondrocytes. β-catenin could be a target of DDH treatment by recovering the chondrogenic ability caused by LRP1 deficiency.

## Supplementary Material

Supplementary File

## Data Availability

All study data are included in the article and/or *SI Appendix*.
