# Supplementary material for: Heterozygous LRP1 deficiency causes developmental dysplasia of the hip by impairing triradiate chondrocytes differentiation due to inhibition of autophagy
Source: Proc Natl Acad Sci U S A. 2022 Sep 6;119(37):e2203557119. doi: 10.1073/pnas.2203557119 (PMC9477389; doi:10.1073/pnas.2203557119)
Supplement: Supplementary File [file pnas.2203557119.sapp.pdf]

Supplemental Materials

Supplemental Table 1. Clinical information of the DDH patients carrying *LRP1* variants.

Supplementary Fig. 1. Information and radiographic features of the DDH patients carrying *LRP1* variants.

Supplementary Fig. 2. *LRP1* variants in DDH. a) Pedigrees and Sanger sequencing results of two families carrying heterozygous *LRP1* missense variants. The position of each variant is indicated by an arrow in the sequence chromatogram. (b) Amino acid alignments in different species around the missense variant. p.R1783 and p.T2129 are highly evolutionarily conserved. (c) *LRP1* variants identified in non-familial DDH patients.

Supplementary Fig. 3. Data on the *Lrp1* model mice. a) Sanger sequencing of heterozygous and homozygous *Lrp1* ^{R1783W} mice their wild-type littermates (WT). b) Western blot for Lrp1. Hip articular tissues were isolated from heterozygous (HET) and homozygous (HOM) *Lrp1* ^{R1783W} mice and *Lrp1*$^{+/-}$ mice, and their WT littermates at age 4 weeks. WT1 and WT2 are littermates of KI and *Lrp1*$^{+/-}$ mice, respectively. Relative protein levels were determined by a density analysis. Values are means ± SD for 3 independent experiments (n = 9). *p < 0.05; **p < 0.01; ***p < 0.0001.

Supplementary Fig. 4. Acetabulum of *Lrp1*^{R1783W} mice and their wild-type littermates at 8 weeks. Homozygous KI and heterozygous KI mice showed similar views with their WT littermates.

Supplementary Fig. 5. The Alizarin red staining and Alcian blue staining of ADTC5 after the chondrogenic differentiation induced with 1% ITS for 7 days. The positive results of Alcian blue staining and negative results of Alizarin red staining indicating that the cells differentiated into chondrocytes under the induction of chondrogenic differentiation medium. Scale bar for 4x: 500 μm, for 40x: 50 μm

Supplementary Fig. 6. Western-blots for levels of autophagy and β-catenin in chondrogenesis induced ADTC5 cells on day 1, 4, 7, 10, and 12.

Supplementary Fig. 7. Western-blots for levels of upstream proteins (p-GSK3β, Lrp6, Dvl2 and GSK3β) of β-catenin in Wnt signaling.

Supplement Table 1. Clinical information of the DDH patients carrying *LRP1* variants.

Patient No.	Age	Site	Acetabulum index	
			Left	Right
2621	2 years 1 month	Bilateral	43	40
2726	1 year 5 months	Left	34	28
3193	5 months	Right	25	30
3151	4 months	Left	42	27
3211	6 months	Right	28	32
3196	8 months	Left	37	26
3161	1 year 8 months	Bilateral	30	33
3174	1 year	Right	31	41
3210	1 year 6 months	Bilateral	32	34

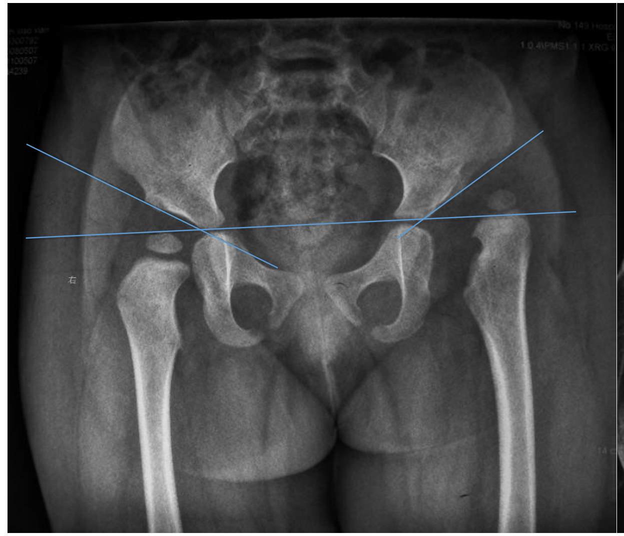

2621

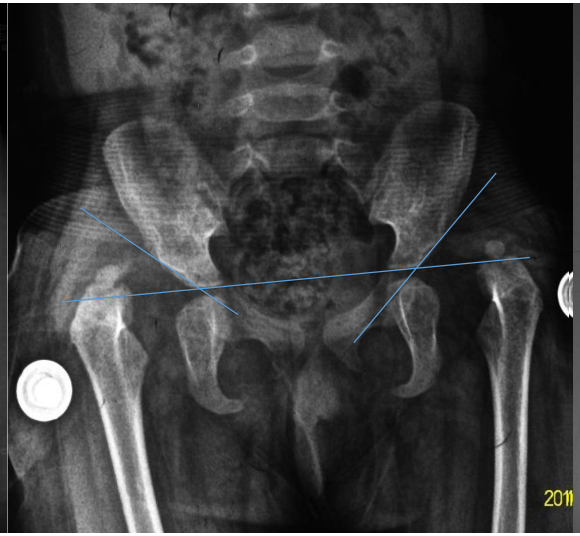

2726

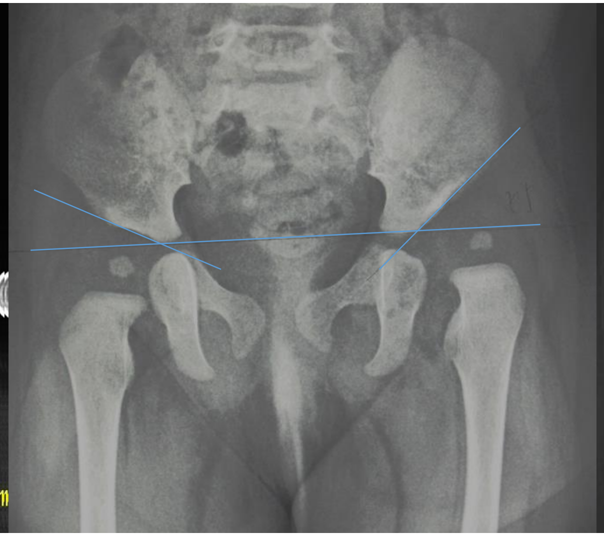

3151

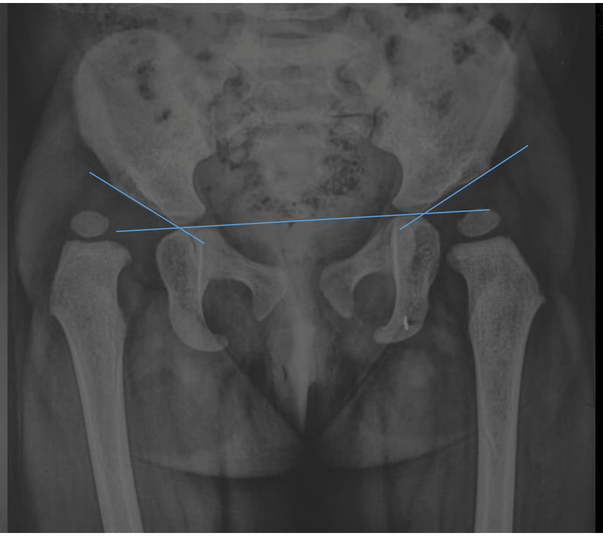

3161

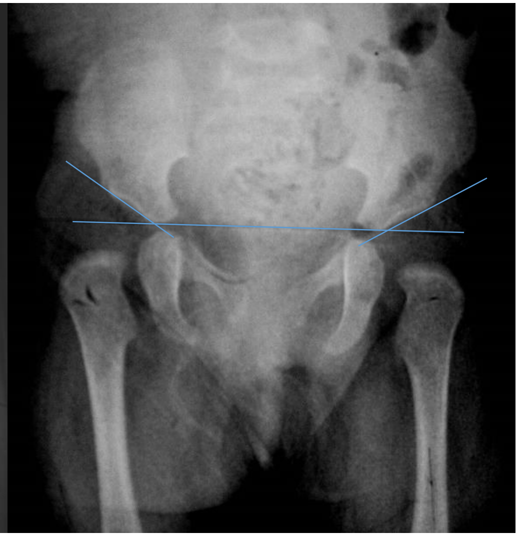

3211

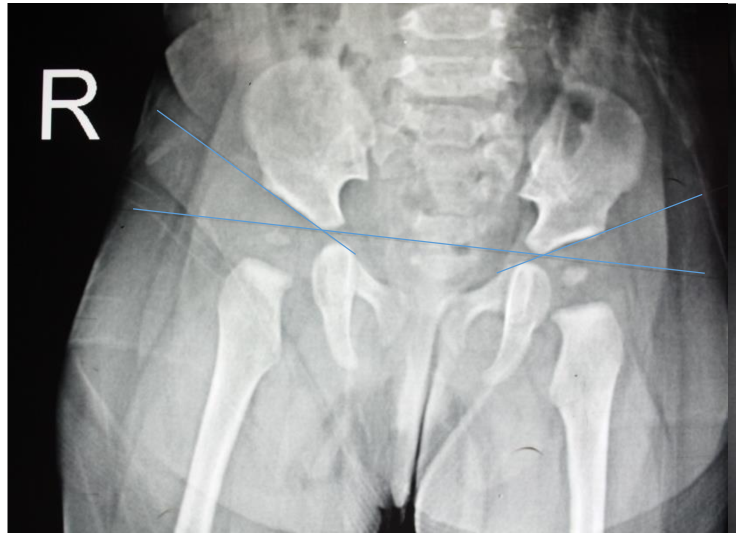

3193

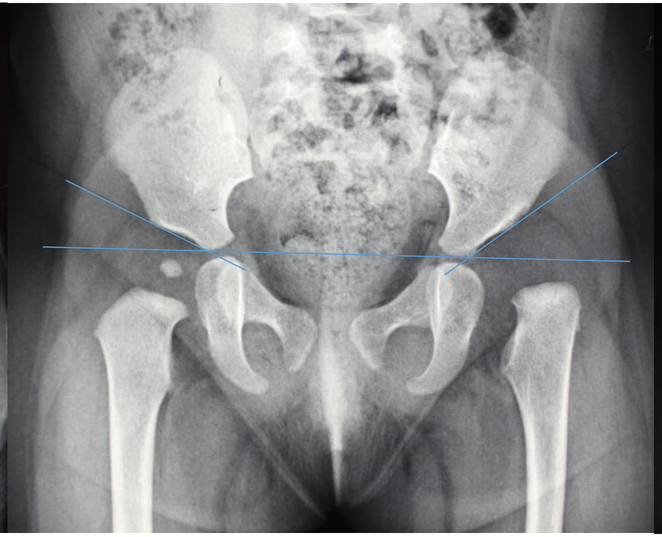

3196

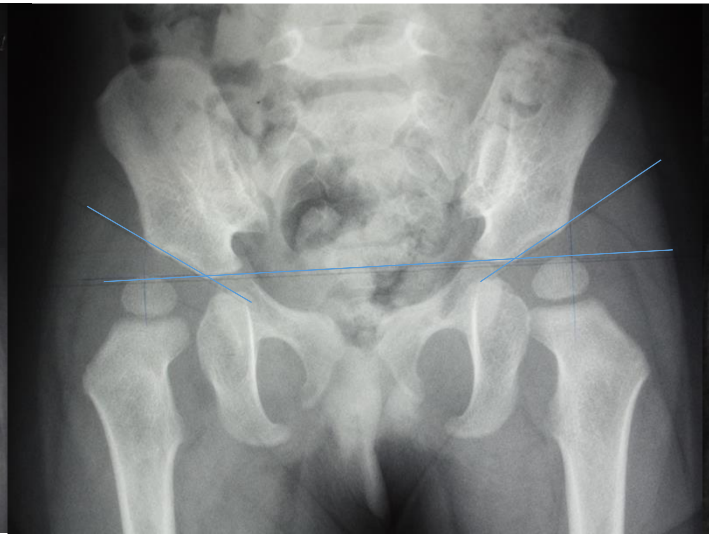

3210

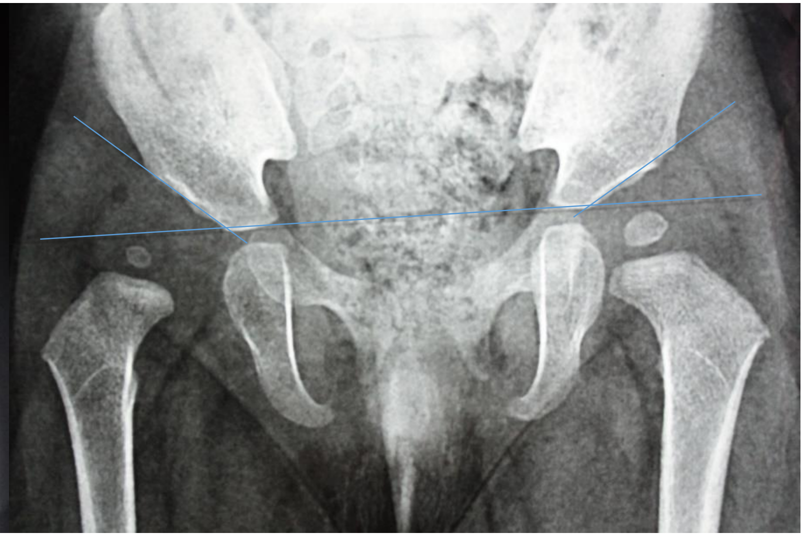

3174

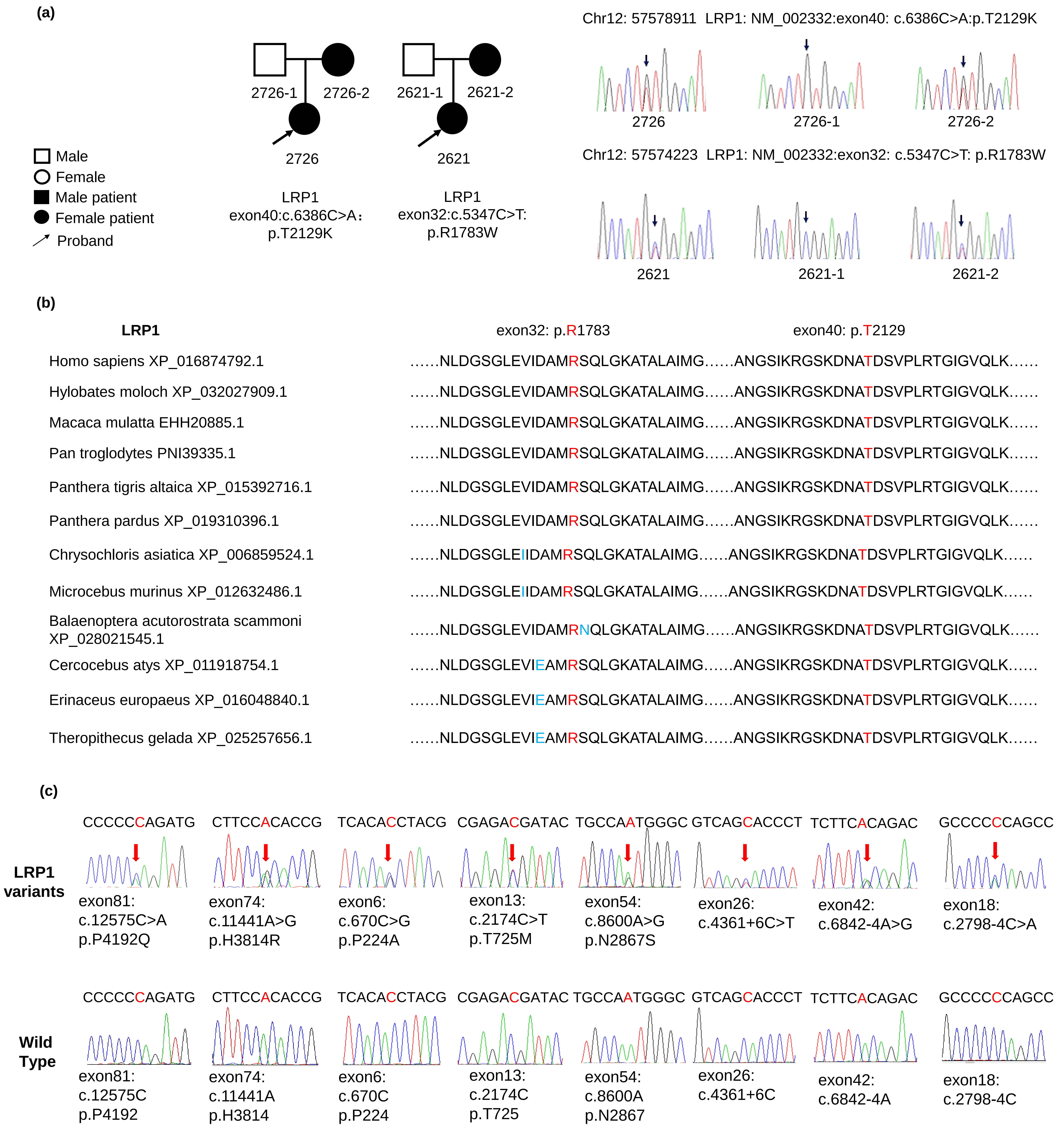
(a)
Chr12: 57578911 LRP1: NM_002332:exon40: c.6386C>A:p.T2129K
2726-1
2726-2
2621-1
2621-2
2726
2621
2726
2726-1
2726-2
Male
Female
Male patient
Female patient
Proband
Chr12: 57574223 LRP1: NM_002332:exon32: c.5347C>T: p.R1783W
LRP1
exon40:c.6386C>A：
p.T2129K
LRP1
exon32:c.5347C>T:
p.R1783W
2621
2621-1
2621-2
(b)
LRP1
exon32: p.R1783
exon40: p.T2129
Homo sapiens XP_016874792.1 ......NLDGSGLEVIDAMRSQLGKATALAIMG......ANGSIKRGSKDNATDSVPLRTGIGVQLK......
Hylobates moloch XP_032027909.1 ......NLDGSGLEVIDAMRSQLGKATALAIMG......ANGSIKRGSKDNATDSVPLRTGIGVQLK......
Macaca mulatta EHH20885.1 ......NLDGSGLEVIDAMRSQLGKATALAIMG......ANGSIKRGSKDNATDSVPLRTGIGVQLK......
Pan troglodytes PNI39335.1 ......NLDGSGLEVIDAMRSQLGKATALAIMG......ANGSIKRGSKDNATDSVPLRTGIGVQLK......
Panthera tigris altaica XP_015392716.1 ......NLDGSGLEVIDAMRSQLGKATALAIMG......ANGSIKRGSKDNATDSVPLRTGIGVQLK......
Panthera pardus XP_019310396.1 ......NLDGSGLEVIDAMRSQLGKATALAIMG......ANGSIKRGSKDNATDSVPLRTGIGVQLK......
Chrysochloris asiatica XP_006859524.1 ......NLDGSGLEIIDAMRSQLGKATALAIMG......ANGSIKRGSKDNATDSVPLRTGIGVQLK......
Microcebus murinus XP_012632486.1 ......NLDGSGLEIIDAMRSQLGKATALAIMG......ANGSIKRGSKDNATDSVPLRTGIGVQLK......
Balaenoptera acutorostrata scammoni XP_028021545.1 ......NLDGSGLEVIDAMRNQLGKATALAIMG......ANGSIKRGSKDNATDSVPLRTGIGVQLK......
Cercocebus atys XP_011918754.1 ......NLDGSGLEVIEAMRSQLGKATALAIMG......ANGSIKRGSKDNATDSVPLRTGIGVQLK......
Erinaceus europaeus XP_016048840.1 ......NLDGSGLEVIEAMRSQLGKATALAIMG......ANGSIKRGSKDNATDSVPLRTGIGVQLK......
Theropithecus gelada XP_025257656.1 ......NLDGSGLEVIEAMRSQLGKATALAIMG......ANGSIKRGSKDNATDSVPLRTGIGVQLK......
(c)
LRP1 variants
CCCCCCAGATG
CTTCCACACCG
TCACACCTACG
CGAGACGATAC
TGCCAATGGGC
GTCAGCACCCT
TCTTCACAGAC
GCCCCCCAGCC
exon81: c.12575C>A p.P4192Q
exon74: c.11441A>G p.H3814R
exon6: c.670C>G p.P224A
exon13: c.2174C>T p.T725M
exon54: c.8600A>G p.N2867S
exon26: c.4361+6C>T
exon42: c.6842-4A>G
exon18: c.2798-4C>A
Wild Type
CCCCCCAGATG
CTTCCACACCG
TCACACCTACG
CGAGACGATAC
TGCCAATGGGC
GTCAGCACCCT
TCTTCACAGAC
GCCCCCCAGCC
exon81: c.12575C p.P4192
exon74: c.11441A p.H3814
exon6: c.670C p.P224
exon13: c.2174C p.T725
exon54: c.8600A p.N2867
exon26: c.4361+6C
exon42: c.6842-4A
exon18: c.2798-4C

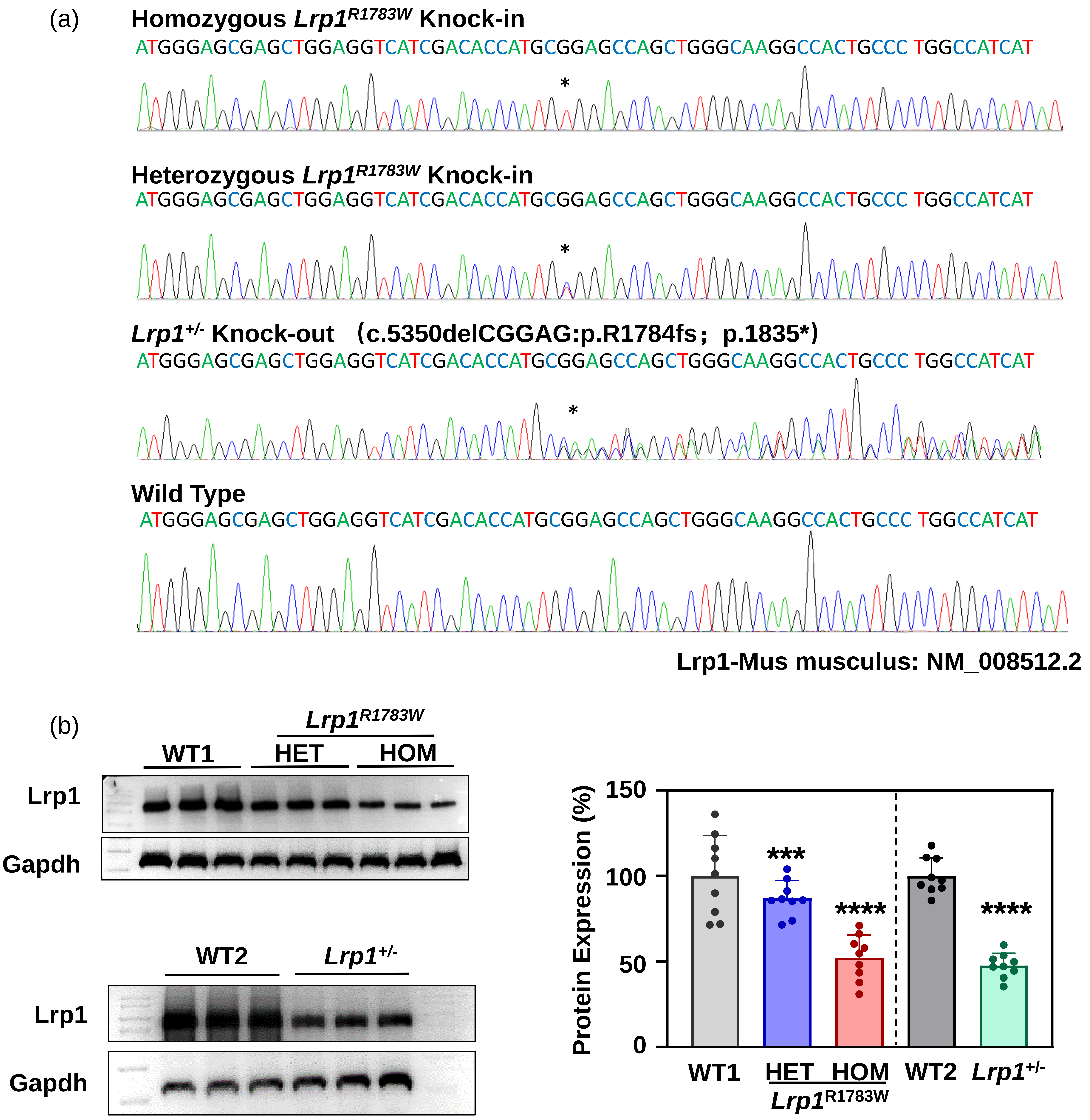
(a)
Homozygous Lrp1R1783W Knock-in
ATGGGAGCGAGCTGGAGGTCATCGACACCATGCGGAGCCAGCTGGGCAAGGCCACTGCCC TGGCCATCAT
Heterozygous Lrp1R1783W Knock-in
ATGGGAGCGAGCTGGAGGTCATCGACACCATGCGGAGCCAGCTGGGCAAGGCCACTGCCC TGGCCATCAT
Lrp1+/- Knock-out （c.5350delCGGAG:p.R1784fs；p.1835*）
ATGGGAGCGAGCTGGAGGTCATCGACACCATGCGGAGCCAGCTGGGCAAGGCCACTGCCC TGGCCATCAT
Wild Type
ATGGGAGCGAGCTGGAGGTCATCGACACCATGCGGAGCCAGCTGGGCAAGGCCACTGCCC TGGCCATCAT
Lrp1-Mus musculus: NM_008512.2
(b)
Lrp1R1783W
WT1
HET
HOM
Lrp1
Gapdh
WT2
Lrp1+/-
Lrp1
Gapdh
Protein Expression (%)
150
100
50
0
***
****
****
WT1
HET
HOM
Lrp1R1783W
WT2
Lrp1+/-

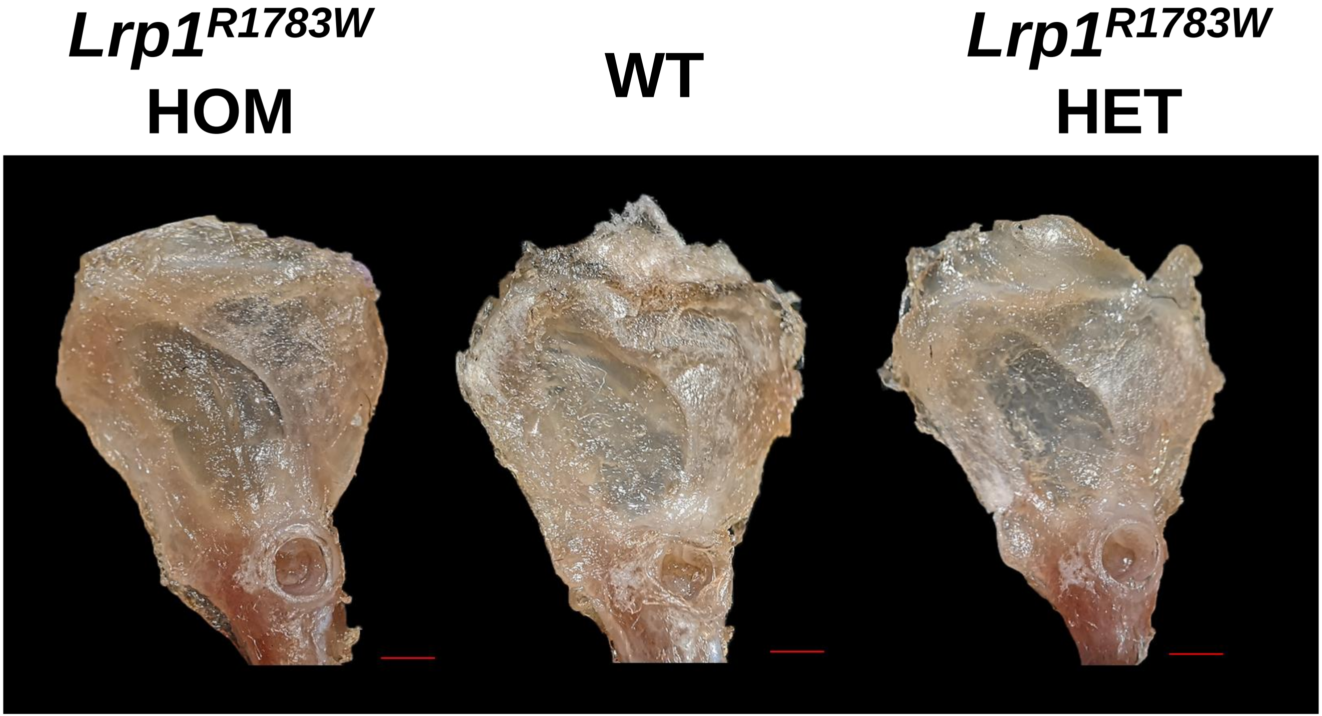
$Lrp1^{R1783W}$ HOM
WT
$Lrp1^{R1783W}$ HET

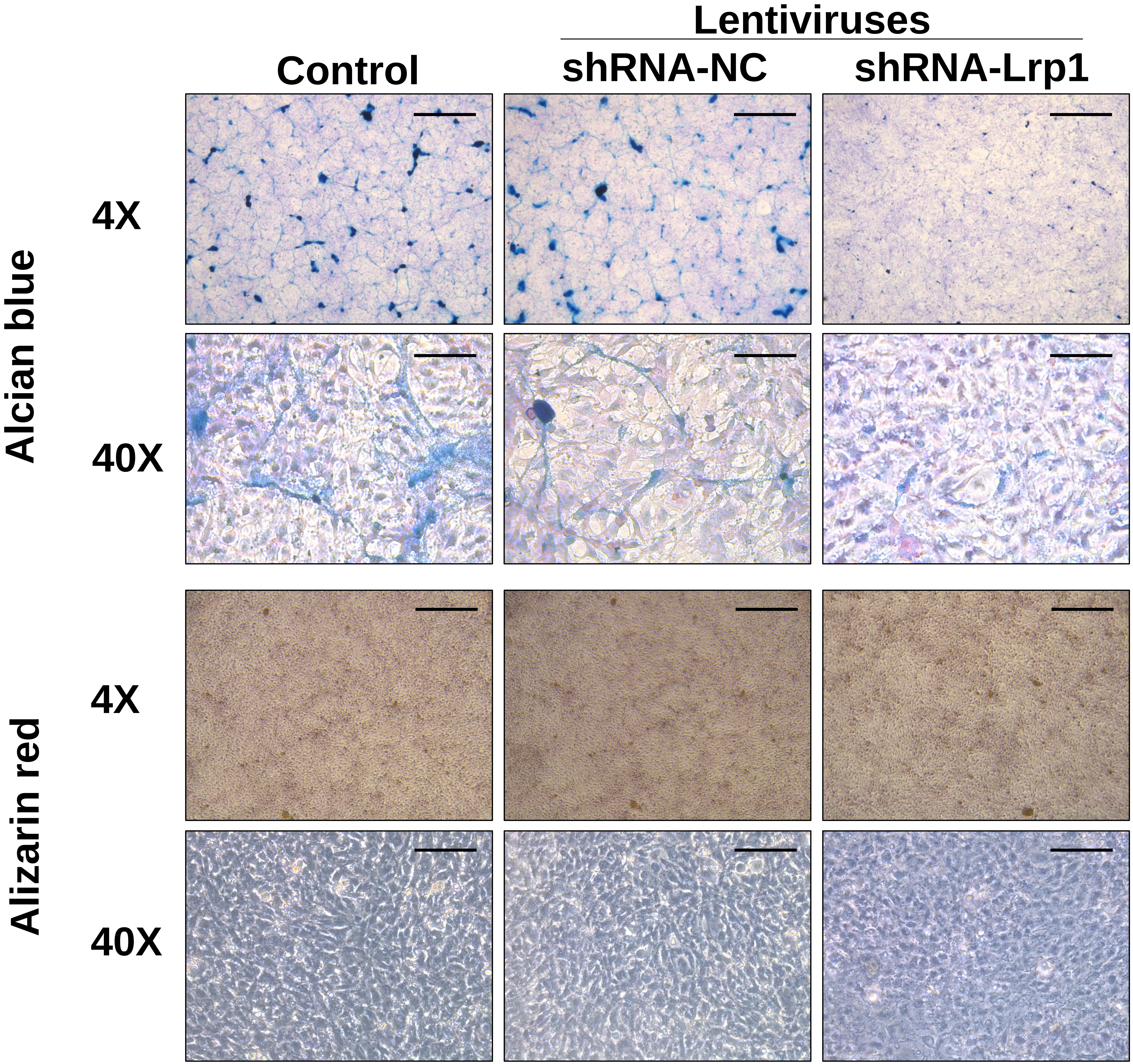
Lentiviruses
Control
shRNA-NC
shRNA-Lrp1
Alcian blue
4X
40X
Alizarin red
4X
40X

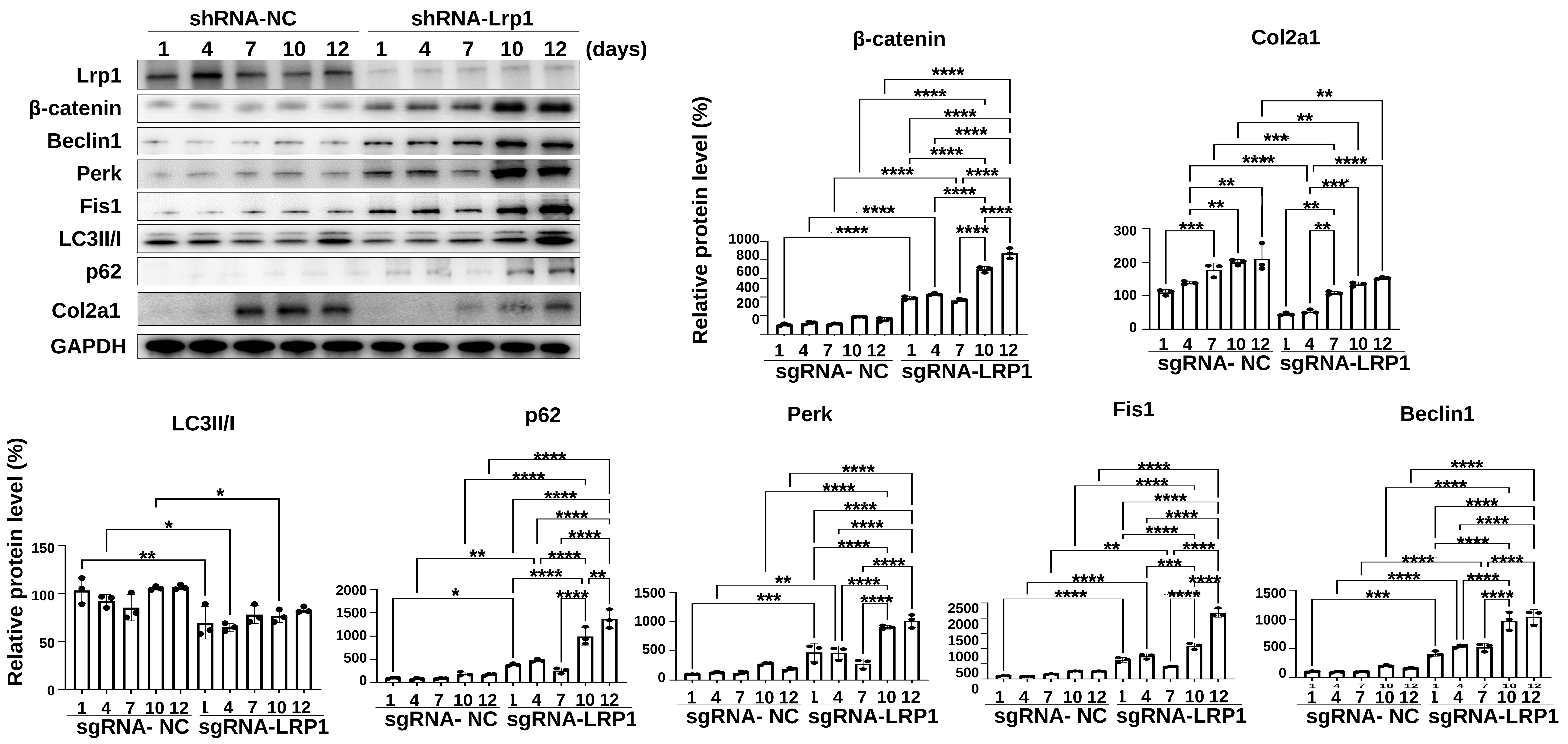
shRNA-NC
shRNA-Lrp1
1 4 7 10 12 1 4 7 10 12 (days)
Lrp1
β-catenin
Beclin1
Perk
Fis1
LC3II/I
p62
Col2a1
GAPDH
β-catenin
Col2a1
LC3II/I
p62
Perk
Fis1
Beclin1
Relative protein level (%)
sgRNA- NC
sgRNA-LRP1
1 4 7 10 12
0 200 400 600 800 1000
0 100 200 300
0 50 100 150
0 500 1000 1500 2000
0 500 1000 1500
0 500 1000 1500 2000 2500
0 500 1000 1500
*
**
***
****

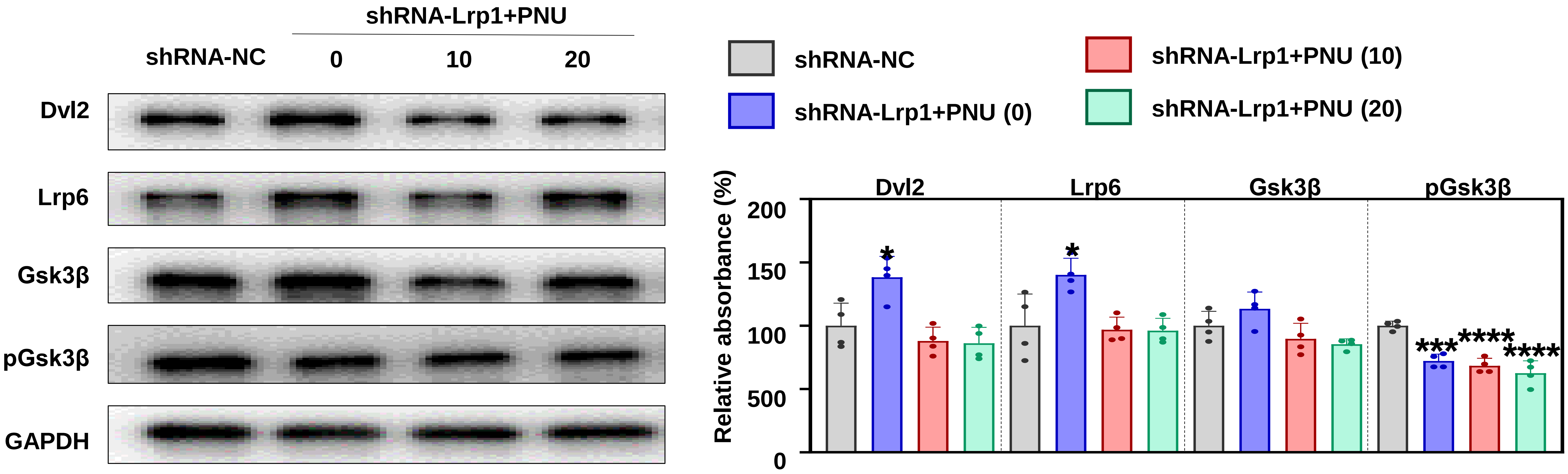

shRNA-Lrp1+PNU
shRNA-NC
0
10
20
Dvl2
Lrp6
Gsk3β
pGsk3β
GAPDH
shRNA-NC
shRNA-Lrp1+PNU (0)
shRNA-Lrp1+PNU (10)
shRNA-Lrp1+PNU (20)
Relative absorbance (%)
200
150
100
500
0
Dvl2
Lrp6
Gsk3β
pGsk3β
*
*
***
****
****
